# Reasonable adjustments for autistic clinicians: A qualitative study

**DOI:** 10.1371/journal.pone.0319082

**Published:** 2025-03-25

**Authors:** Helen Smith, Sebastian C. K. Shaw, Mary Doherty, Jonathan Ives

**Affiliations:** 1 Centre for Ethics in Medicine, Bristol Medical School, University of Bristol, Bristol, United Kingdom; 2 Department of Medical Education, Brighton and Sussex Medical School, Brighton, United Kingdom; 3 University College Dublin, School of Medicine, Dublin, Ireland; Children’s Hospital Affiliated of Zhengzhou University: Zhengzhou Children’s Hospital, CHINA

## Abstract

Autistic people experience barriers to accessing healthcare. Autistic clinical professionals may be able to help improve this situation. Previous research, however, has shown that Autistic clinical professionals experience numerous challenges in the workplace. If there is a ‘substantial’ and ‘long-term’ negative effect on the person’s ability to do normal daily activities, then Autism may be considered a disability under The Equality Act 2010; the jurisdiction of which covers Great Britain. Autistic clinical professionals working in healthcare settings across England, Wales, and Scotland are therefore entitled to reasonable adjustments to aid them in their clinical practice. This is a qualitative study. We recruited 82 Autistic clinical professionals via social media to complete an online survey. Questions broadly explored: 1) the challenges they faced in their clinical workplaces; and 2) the reasonable adjustments that they needed, had, or needed but did not have. Data were analysed quasi-thematically, also drawing on the principles of content analysis. Respondents reported multiple challenges from our analysis, from which we developed 8 themes: gaining and attending employment, reasonable adjustments under the radar, connecting and integrating (specifically, the communication mismatches between Autistic professionals and non-autistic colleagues, and fitting in socially and professionally), executive functioning, change, working environment, working practices/cultures, and the consequences and effects on Autistic clinical professionals). We recommend that Autistic clinical professionals and their employers individually discuss and iteratively revisit the unique combination of reasonable adjustments suitable for each person. In this way, employers may provide equitable workplaces for their staff which will benefit not only them, but their patients, and healthcare as a whole.

## Introduction

Life experiences are inherently tied to one’s fit – within environment, social group, and broader culture [1]. When there is a mismatch between the person and these external factors, it opens the door to challenging experiences [2]. This is particularly evident in the case of minority groups, as society has developed to suit the majority [1]. Being Autistic is no exception to this. After all, to be Autistic is, by definition, to experience a particular pattern of differences compared to the neurotypical majority. More specifically, autism can be defined “as a lifelong neurodevelopmental difference that influences the way a person interacts and communicates with others and experiences the world around them” [2]. Recognising this mismatch being key to any disablement is central to the neurodiversity paradigm, refuting more historical deficit perspectives that focus on inherent impairments within Autistic people [3].

The United Kingdom’s National Institute for Healthcare Excellence estimates Autism prevalence in adults at about 1.1% [4]. However, evidence from the United States suggest a higher prevalence of around 1 in 36 people being Autistic [5].

Given the challenges experienced by Autistic people, due to the aforementioned mismatches, being Autistic typically can be considered a disability as per the Equality Act of 2010 in Great Britain [6]. This requires employers to implement reasonable adjustments in an effort to ameliorate external barriers – recognising both the contextual nature of disablement and the importance of changing external factors in response.

The UK Advisory, Conciliation and Arbitration Service (ACAS) defines ‘reasonable adjustments’ as “changes an employer makes to remove or reduce a disadvantage related to someone’s disability” [7]. If an employer knows or should reasonably have known that their employee is disabled, then they must make reasonable adjustments [6,7]. The adjustment would need to reduce or remove the disadvantage of the person’s disability whilst being practical, affordable, and not harming the health or safety of others [7]. However, the term ‘reasonable’ is vague and unique to each employee-need and employer-resource combination; some larger employers (e.g., the United Kingdom’s National Health Service) would have greater resources available to make adjustments for individuals than others (e.g., a small independently owned cake shop). Employment tribunals step in when no agreement can be reached between employer and employee as to the sufficiency of the adjustments made (or not as the case may be) [7].

There is a requirement that registered healthcare professionals’ health is sufficient for them to be ‘fit to practise’. If a registrant’s (i.e., a healthcare professional holding registration to practise with their healthcare regulator) fitness to practise is called into question, their professional regulator can investigate them via a fitness to practise procedure [8]. Both the General Medical Council and the Nursing and Midwifery Council are clear that no disability is, in itself, a fitness to practise issue [9,10]. Despite this, there is precedent for a doctor being removed from their job due to receiving and disclosing an autism diagnosis [11].

There is some evidence to suggest that Autistic clinical professionals, i.e., those healthcare professionals who observe and treat patients, receive little in the way of reasonable adjustments [12]. In a cross-sectional study of the experiences of Autistic doctors, 29% had not disclosed their being Autistic in the workplace [13]. This non-disclosure may come at a personal cost, prohibiting access to formally implemented reasonable adjustments. The same study, however, found that only 49% of those who had asked for reasonable adjustments at work had received them, and that those requesting and not receiving them experienced the highest rates of self-harm [13]. Furthermore, nearly half of respondents reported that just being able to be open about being Autistic in their workplace was a desirable reasonable adjustment in itself [13].

When we commenced this study there were no other studies that had explored this in other Autistic clinical professionals, nor in further depth – what reasonable adjustments were made for them, what their needs were, or what the barriers were to reasonable adjustment implementation. Since analysing our data, Curnow *et al.* [14] have provided a very useful analysis of reasonable adjustment needs based on qualitative interviews of 34 autistic people who work in professional roles within health and education in Scotland. Their report was drafted with input from management and human resources professionals and offers guidance regarding improvements in training, recruitment, and employment for Autistic health professionals. Their key messages were:

- Embrace neurodiversity,
- Understand masking,
- Provide safe and trusting environments for disclosure,
- Support self-advocacy and shared advocacy, and
- Provide and maintain reasonable adjustments and accommodations.

These key messages offer a strong starting point for understanding and supporting Autistic employees as well as guidance on how to create a neurodiversity-affirming work environment. Our study examines similar terrain, but with a focus solely on healthcare professionals – and not just in Scotland, but throughout Great Britain. We used a survey methodology that allowed us to access a greater number of participants, including those Autistic clinical professionals who might not be comfortable taking part in interviews. Our paper seeks to answer the following research questions:

- *What reasonable adjustment needs are experienced by Autistic clinical professionals practicing in Great Britain?*
- *What reasonable adjustments are in place for Autistic clinical professionals practicing in Great Britain?*

## Methods

### Study conception and positionality

This study benefits from a mixed insider-outsider research team, aligning with the neurodiversity paradigm [15], and draws on representation from more than one health discipline. Author 1 is a multiply neurodivergent person (including Autistic), and a nurse registered with the Nursing and Midwifery Council. Author 2 is an Autistic doctor, working in Great Britain. Author 3 is an Autistic doctor practising in Ireland. Author 4 is an expert empirical bioethicist who does not identify as Autistic.

Author 1 was the driving force behind all stages of this project from conception to completion. In terms of accounting for reflexivity in this work, Author 1 drew from their own historical experiences of working as an Autistic clinical professional to the benefit of this project, however they were aware of the limitations of their own singular experience as a nursing registrant and their fear of accidently misrepresenting their Autistic peers. In response to this, a steering committee was formed (Author 2 and Author 3) and an expert empirical bioethicist brought aboard (Author 4). The whole team were involved throughout, helping to guide and steer the project from conception to write up. This permitted constructive critique to strengthen this research and reduce the impact of individual bias.

Our cross-disciplinary practical experience of the working conditions of Autistic clinical professionals in Great Britain allowed for additional insight into the questions that needed to be asked, empathetic analysis of the answers, and the hope of bringing epistemic empowerment and justice to our neurokin. Of course, this carries the risk of bringing our own internal biases into this work. To counterbalance this, part of Author 4’s role was to act as an outsider and critical friend, and challenge the interpretation of the data. Additionally, the inclusion of three researchers with different lived experiences of being an Autistic clinical professional helped mitigate individual bias.

### Methodology and philosophical alignment

This is a qualitative study, with elements of quantitative thematic reporting, in which we take a broadly critical realist perspective. This aligns with a position articulated by Ives [16], which takes a pragmatic view, assuming an epistemological scepticism that ‘admits the possibility of...truth but holds that we do not currently have the tools to recognise or demonstrate it’. As such we see this work as moving towards better accounts of experience, accepting that any knowledge claims generated are *prima facie* constructed in the research encounter, but acknowledging they might nonetheless track reality. This is consistent with measures taken (such as triangulating analysis between Author 1 and Author 4) to reduce overt bias. Our analytic aim was to clearly and unambiguously represent views and experiences without adding layers of thematic interpretation that might impede authentic presentation of our respondents.

### Data collection, participants and sampling

We used a cross-sectional survey to gather data regarding existing or desired reasonable adjustments for Autistic clinical professionals, in which quantitative and qualitative data were acquired more-or-less simultaneously from a specified population [17] of Autistic clinical professionals. We decided a survey was the most appropriate format to set the bar for inclusion as low as possible over a short (one-month) recruitment period. Using a survey ensured the largest amount of flexibility for participants to partake (many of whom work shifts, thereby preventing in-person data collection), to answer the questions as comprehensively as they wished to (without pressure of time), and avoided the additional social burden of enduring the more traditional synchronous interview conditions.

These practical considerations are theoretically aligned with our epistemological position, as we have aimed to authentically represent participant experiences with minimal interpretation beyond what is necessary for thematic representation.

The survey tool was initially developed by Author 1 with input from the wider team. This included trial runs within the team to consider face and content validity, leading to refinements (e.g., in the phrasing of questions and the order of answers to multiple-choice questions) being made before finalisation and deployment. Supporting information S1 File outlines the survey questions asked of participants.

Our target participants were:

- Autistic: We did not require participants to have a formal diagnosis of autism to enrol in this study. Formal diagnosis of autism is a privilege and not something that is available to all who are Autistic. There are multiple significant barriers to diagnosis; for example, the assessing clinician’s biased perception that autism is a ‘boy’s disorder’ [18] creates a barrier for individuals whose gender is not male when being assessed for autism. A diagnosis of autism is not required for the Equality Act to apply, only that there is evidence that the person’s difficulties are chronic and substantial [19].
- currently registrant with the General Medical Council, Nursing and Midwifery Council, or the Health & Care Professions Council;
- currently employed in clinical practice and working within the scope of their registration;
- currently employed in Great Britain (England, Wales, or Scotland).

Purposive sampling [17] was used to recruit our target population. We utilised social media advertising via Twitter/X as well as approaching relevant organisations and the administration teams of peer support groups populated with Autistic people and asking them to post the link to our survey on Facebook and/or to re-tweet the advert.

The advert linked to a study webpage containing further study information and a participation link. The survey was constructed in Microsoft Forms, hosted on the University of Bristol’s secure servers, and open for one month: 20 October 2022 through to 17 November 2022.

A participant information sheet was provided, and informed consent gained at the start of the survey and confirmed on submission. All data were anonymous, and only completed surveys entered the dataset.

To enable participants free expression of the fullness of their identities and characteristics we provided open-text boxes for all demographic data [20], apart from age, regulator selection, and diagnosis status.

At the point of study design and data collection we were unaware of the growing concerns about potential ‘scammer’ participants in online autism research, especially where a financial incentive is involved, and the research is advertised online [21]. We did not practice any techniques to ensure that participants were genuine, e.g., checking IP addresses; however, we do not believe that this project was affected by this issue as we offered no financial incentive to participate and felt participants’ accounts to be convincingly Autistic and representative of the experiences of healthcare professionals.

### Ethics

This study was approved by the University of Bristol’s Faculty of Health Sciences Research Ethics Committee (FREC) Ref: 12134. A participant information sheet was provided, and informed consent gained at the start of the survey and confirmed on submission.

This study adopted relational ethical considerations [22]. For example, unless vital for contextual understanding, we chose to not identify participants’ professional group/role in our reporting – firstly to protect participants from the risk of identity triangulation and secondly to avoid inference being drawn about subgroups, which our data do not have the numbers to support.

## Analysis

In keeping with our analytic aims, analysis of qualitative survey data was informed by the six phases of Reflexive Thematic Analysis [23,24], and by Hsieh & Shannon’s [25] conventional content analysis which enables a more straightforward representation of the data without additional layers of reflexive thematic interpretation. Our aim was to reduce misinterpretation to fairly and unambiguously represent the data [24]. This required us to carefully map the content of answers, and then group them into descriptive themes for reporting. Informed by Braun & Clarke [23,24] and Hsieh & Shannon’s [25] approaches, this involved inductively developing descriptive themes through systematic engagement with the dataset.

The following outlines our actions:

1. *Familiarisation of data*: Author 1 went through the entire dataset line-by-line, determining and assigning codes to each quote, and mapping these to a table in Microsoft Word.
2. *Generation of codes:* Once all quotes had been coded, Author 1 checked every quote to ensure that each matched their code and code descriptions. Codes and code descriptions were then refined overall to ensure that each reflected the quotes that they represented.
3. *Combining codes into themes*: Author 1 then collapsed the codes into themes through pattern-matching; i.e., codes which reflected related experiences were grouped together.
4. *Reviewing themes*: Author 4 checked and double coded around 10% of the codes. All authors were then invited to review the coding and themes. Refinements to the coding and themes (e.g., refinements of the descriptors given to themes) were then made.
5. *Determine significance of themes*: Author 1 formulated the first draft of this report, during which they constructed a narrative to the data by organising the themes so that they told a clear story: from the point that an Autistic clinical professional seeks work, through to the challenges that they face once in work. The author team discussed the importance of the themes presented in this draft; all themes were retained and presented in this report so as to demonstrate the variety of challenges that Autistic clinical professional face.
6. *Reporting of findings*: After multiple rounds of drafting, this manuscript is our final report.

This entire process was additionally informed by the experiences of the Autistic authors who drew on their own experiences of being Autistic clinical professionals themselves to offer insight into the meaning and significance of participants’ responses.

We were informed by Braun and Clarke’ stepwise process, but deviated from them in terms of interpretation and going beyond the data; here we were influenced by the conventional content analysis approach as per Hsieh and Shannon [25]. This approach is similar to Braun and Clarke in that pre-conceived categories are avoided when arranging the data, and that categories and names for categories flow from the data itself. Conventional content analysis promotes immersion in the data which allowed us to recognise insights from it and to describe the phenomenon that is the Autistic clinical professional experience in the workplace. This means that our analysis is a more straightforward representation of the data than would be typical when using Braun and Clarke’s approach; i.e., we have presented the data *as it is* rather than having added extra layers of reflective analysis. Our aim was to articulately and accessibly inform readers of the variety of specific challenges that arise for Autistic people in their workplaces – using their own language. We wanted to be as true to our participants’ authentic voices as possible.

## Results

Two respondents identified as students and so did not meet the inclusion criteria, and as such their data were removed. This left a cohort of 82 participants. A brief overview of the respondent demographics is offered in Table 1.

**Table 1:** Brief overview of responder demographics.

**Sexual orientation:** Heterosexual – 53 (64.63%) Not heterosexual – 16 (19.51%) N/A - 13 (15.85%) Total = 82	**Ethnicity:** Arab – 1 (1.21%) Asian – 1 (1.21%) Asian-British – 2 (2.43%) Chinese – 2 (2.43%) Indian – 1 (1.21%) Pakistani – 1 (1.21%) Mixed/ mixed ethnicity – 3 (3.65%) White – 63 (76.82%) British/UK - 5 (6.09%) N/A – 3 (3.65%) Total = 82	**Age ranges:** 18-29 – 8 (9.75%) 30-39 – 31 (37.80%) 40-49 – 28 (34.14%) 50-59 – 12 (14.63%) 60-69 – 3 (3.65%) Prefer not to say – 1 (1.21%) Total = 82
**Gender:** Male – 15 (18.29%) Female – 59 (71.95%) Non-binary – 4 (4.87%) N/A – 4 (4.87%) Total = 82	**Registered with which regulator?:** General Medical Council – 59 (71.95%) Nursing and Midwifery Council – 13 (15.85%) Health and Care Professions Council– 10 (12.19%) Total = 82	**Diagnosis:** Formal – 65 (79.26%) Self-diagnosed – 17 (20.73%) Total = 82
**Employer:** Public healthcare provider (e.g., NHS) – 71 (86.58%) Private healthcare provider (e.g., private hospital) – 2 (2.43%) Other (e.g., self-employed) – 9 (10.9%) Total = 82		**Workplace environments:** Primary care – 13 (15.85%) Community care – 16 (19.51%) Secondary care – 36 (43.90%) Primary and secondary care – 2 (2.43%) Tertiary care – 11 (13.41%) Local Authority – 1 (1.21%) Civil service – 1 (1.21%) Across all sectors – 1 (1.21%) Pre-hospital – 1 (1.21%) Total = 82
*Have you asked for reasonable adjustments from your employer?* •Yes - 44 (53.65%) • No - 38 (46.34%)		
***Reasonable adjustments associated with being Autistic agreed with employer:*** Yes – 28 (34.14%) No – 54 (65.85%)		

This study attracted participants from a cross-section of clinical disciplines, professions, and workplaces. We noted the larger proportion of female (71.95%) to male (18.29%) and non-binary (4.87%) persons, which was close to being representative to the 68.7% women and 31.3% men reported as employed by NHS England and NHS Improvement’s gender pay gap report in 2022 [26], however the gender pay gap report did not mention those not of the gender binary, so it is difficult to determine true representation in this area. In June 2022, out of NHS staff whose ethnicity was known, 74.3% were white and 25.7% were from ethnic minority groups [27]. White participants came close to being representative of the NHS workforce at 76.82%, but only 13.41% identified themselves as those from ethnicities of the global majority; for this reason, we determine that our project likely underrepresents the richness of diversity from the global majority who are present in the health services of Great Britain. The larger number of doctors who are registered to practice with the General Medical Council (71.95%) as compared to nurses and midwives who are registered to practice with the Nursing and Midwifery Council (15.85%) or health and care professionals registered to practice with the Health and Care Professions Council (12.19%) was, we suspect, due to Author 2&3’s facilitating recruitment via Autistic Doctors International [28], which would have reached more of the medical community than other clinical professional groups.

We asked participants to tell us about their needs leading to requirement of reasonable adjustments, the reasonable adjustments they needed themselves, the reasonable adjustments they actually received, and the reasonable adjustments they did not receive. The headings below present high-level reporting of the subsequent analysis: we report the challenges that Autistic clinical professionals have experienced, and the reasonable adjustments they stated they needed, had, or needed but did not have.

We developed 8 themes, each representing an area of challenge that Autistic clinical professionals face in their workplaces. Quotes used to illustrate our reporting were lightly edited to ensure anonymity for participants. Numbers in curly brackets after quotes indicate the corresponding participant numbers (*n.b.,* participant numbers go above 82 due to the allocation of numbers during testing pre-survey deployment).

A note on the style of our reporting: it is common in thematic analysis to use idioms and metaphors to describe interpreted themes. For accessibility reasons, we attempted to avoid this as some Autistic people may take phrases literally if the meaning is not entirely obvious.

### 1. Gaining and attending employment.

The first hurdles to entering the workplace were identifying and attaining suitable roles.

*“It can take time and a lot of difficulties/stress to find an environment that is suitable for autistic clinicians especially when you don’t know your autistic. Without this recognition it is hard to ensure the right adjustments are in place.” {85}*

Respondents would welcome adjustments for job interviews; for example, being offered written questions in advance, or even workplace trails in lieu of interviews.

Once employment was secured, some respondents needed transport adjustments such as help in obtaining funding for transport to work, presumably via Access to Work (a UK Government scheme whereby disabled applicants may apply for grants to help them get or stay in work [29]). Others took a different approach (presumably to avoid crowded public transport):

*“When starting [specialism] training I asked for placements within cycling distance of my house and although this wasn’t explicitly granted, all my placements are indeed local.” {32}*

### 2. Reasonable adjustments under the radar.

Here, respondents reported they had arranged the adjustments they needed almost by stealth.

*“I am senior enough to advocate for what I need by myself” {7}*

Some had chosen to work in roles that naturally accommodated them, had been able to arrange their own reasonable adjustments, or already had adjustments in place for something else which additionally met their Autistic needs.

*“I recognised my own needs and found a job that can cater to these needs without having to disclose, after 3 years in training without any adjustments which led to increased anxiety and near burnout.” {60}*

*“I get them for adhd” {70}*

*“I think [my specialism] is ideal for [Autistic clinical professionals] because it cuts out a lot of the clinical communication skills stuff and anxiety and emotional stuff you often get around direct patient care” {13}*

### 3. Connecting and integrating.

As noted in the introduction, Autistic people often experience mismatches between themselves and the external environments, social groups, and broader cultures within which they live. In accordance with this, we noticed common threads of challenges that Autistic clinical professionals reported – especially in terms of connecting and integrating into their working environment, which took shape in three overlapping (but still distinct) concepts: communication mismatches between Autistic professionals and non-autistic colleagues, and fitting in socially and professionally.

**3a) *Communication mismatches between the autistic professional and non-autistic colleagues*:** Respondents highlighted differences in the ways that Autistic and non-Autistic people communicate. In the context of busy healthcare work environments, this became particularly challenging.

Issues were generally noted in both receiving and sending information. For example, when receiving information:

*“I struggle with recognising body language and non-verbal communication, particularly in large groups and sometimes have to rely on [colleagues] to interpret for me, this is worse when exhausted/over stimulated” {19}*

When sending information, respondents noted that their intentions could be misunderstood or considered rude by others:

*“If they don’t know why I’ve done something a particular way then ask me, don’t assume things. There is usually perfectly valid reason but might not be obvious to [non-Autistic] others.” {39}*

*“Interactions with some staff members tricky as they say i am too abrupt/direct/rude” {20}*

The data given to us by respondents predominantly spoke of communication issues with other people generally, rather than with, for example, colleagues, patients or both. But there were examples of how communication styles could be less aligned with colleagues (please see *fitting in professionally* below) yet more aligned with patients:

*“I can relate more to my patients than my colleagues.” {16}*

Communication adjuncts were variously received by Autistic clinical professionals. Many found phone calls challenging, others also struggled with video calls:

*“I also find talking on the phone really stressful owing to not having visual cues and having slow auditory processing; I much prefer written communication which causes problems when some colleagues insist on phoning about everything and do not want to discuss anything over email.” {28}*

*“Struggling with video meetings, not sure when to talk” {76}*

*“Group video calls with multiple people are particularly difficult when there is not a consistent quality to the participants audio with people are different distances from the microphone affecting the volume changing without warning when different people speak.” {89}*

Multiple modes of communication were reported to be especially difficult for this participant:

*“Work related communication is never all in one place - to keep up to date you’re expected to manage more than one email address, several WhatsApp groups, Twitter etc. I can’t cope with being contactable all the time so I mute notifications, but when I get back to work there are hundreds of messages to wade through, which again is overwhelming.” {23}*

Various remedies to these challenges were suggested. Clarity was considered key in terms of workplace policies, documentation, roles, and expectations of Autistic clinical professionals. To aid inclusion, respondents suggested that others invite them to contribute in meetings. Whilst each Autistic person’s communication needs will be unique to them, many respondents reported valuing written communication (e.g., email). Similarly, highly structured communication aids, such as using agendas, or standardised approaches to communication such as the World Health Organisation’s Surgical Safety Checklist [30] were welcomed:

*“WHO team brief has been…very helpful” {80}*

**3b) *Fitting in socially*:** Interpersonal interactions such as meeting new people and reading and responding to social behaviour are a common necessity in healthcare workplaces. However, they were not always comfortable for respondents. Informal social interactions, including small talk and attending work social events, raised challenges for many:

*“Mostly difficulties with interpersonal relations, coffee room, “small talk”. Knowing how to participate with team members” {64}*

*“I also don’t do well socially after the first couple weeks and tend to be left out of social events.” {52}*

*“it is a nightmare when someone suggests that the whole team goes for lunch/to socialise after work (for example) and I can’t even get away and have a break without appearing stand-offish or odd; it is important that I integrate into the team and am seen as a good team player who gets along with people. I know colleagues already find me weird/shy because my conversation patterns (though honed over years) are a bit ‘off’ so I don’t want to do anything to make this worse.” {28}*

Respondents reported changing their Autistic behaviour to aid fitting into neurotypical surroundings; a practice known as ‘masking’ [31]:

*“…have to ‘mask’ a lot at work in order to appear normal/professional and friendly towards colleagues.” {28}*

However, doing this was detrimental to them

*“I was made to feel uncomfortable, and asked to change my behaviour and myself to make others comfortable. My mental health deteriorated, as well as being burnout [sic] and exhausted. At the end, I was ostracised and isolated from my peers at the workplace.” {84}*

**3c) *Fitting in professionally*:** The requirement to fit in professionally created an unavoidable formal dimension to social demands which fostered feelings of unease. Respondents reported being exposed to the pressures of dealing with lots of people in the course of their work and the challenges (e.g., conflict) that each encounter may present:

*“I struggle to cope with angry patients” {88}*

Navigating teamworking, as well as relationships with colleagues and management, could be difficult; yet respondents did not feel this impaired their performance as clinicians:

*“I don’t have problems in consultations, it’s the office politics that I struggle with!” {38}*

*“I don’t seem to struggle with patient interactions but have struggled with [professional] feedback and sometimes in person interactions, particularly with seniors” {12}*

Professional misunderstandings, however, can impact on interpersonal relationships

*“I ask lots of questions which are misinterpreted as me not wanting to do something when really I’m trying to figure out what they want me to do.” {39}*

*“being told I am anxious because I need more detail to understand something” {41}*

*“In the past I have been in trouble due to people perceiving my communication style to be too abrupt.” {65}*

A key reasonable adjustment need was understanding and tolerance of Autistic clinical professionals’ Autistic selves by colleagues and managers.

*“More understanding of the condition, and that it is not a ‘choice’ or something that you can just ‘deal with - I cannot work in a noisy environment, whether I make an effort or not. It is not optional. ‘” {66}*

*“Understanding that high achievers can be autistic.” {9}*

Respondents felt that understanding of Autistic clinical professional team members would be enhanced by staff training and the opportunity for Autistic clinical professionals to regularly check in with seniors:

*“[H]aving a manager who understands autism, having regular meeting with my manager so things can be resolved before they become a big problem for me.” {87}*

### 4. Executive functioning.

Executive functioning refers to the “skills [and] mental processes that enable us to plan, focus attention, remember instructions, and juggle multiple tasks successfully.” [32]. Reported differences in this area were heavily grounded in being considered deficits by our respondents. The general message that the data gave us was that there is a difference in executive functioning which led to particular challenges, which included:

- Multitasking – *“I find multitasking difficult especially when it is quite intense and time bound like while [doing] on calls looking after unwell patients in all wards plus clerking at the same time. I was able to do either of two [but] rather providing any support I was harshly criticised and was put to internal fitness to practice causing me tremendous amount of stress and further lack of confidence” {33}*
- Difficulty managing interruptions – *“being constantly interrupted was very challenging for me.” {65}*
- Longer learning time – *“Lots of things seem to come logically and easily to others but I have to learn them. Learning for me requires sitting and writing notes and preferably a human teaching. That way I came top in many new school exams. But that doesn’t work for a lot of adult learning especially post Covid. I can’t take thorough notes during a speedy webinar and spend hours revising it until I know it all, and I struggle with the fact I don’t remember it all.” {88}*
- Longer processing times – *“I’m slower than my peers in terms of processing information and transitions in work style make this more pronounced (e.g., switching back and forth between face to face and telephone consultations). Being slower is not a favourable trait in the NHS, even if I am more thorough. I think it is viewed as not trying hard enough or not caring which isn’t true.” {52}*
- Organisation – *“Organisation” {85}*
- Task initiation – *“Initiating task[s] can be harder” {85}*
- Task switching – *“I found the...job particularly stressful because I struggle to switch tasks” {65}*
- Time management – *“When it comes to non-clinical work…I have struggles with realising deadlines and encouraging others in my team.” {64}*
- Prioritisation – *“Executive dysfunction meaning I prioritise differently and am told off for it” {42}*
- Poor working memory – *“Poor working memory means I often have to rely on writing out/printing things.” {44}*
- Issues with self-regulation – *“Having to sit still in meetings” {16}*
- Attention issues – *“[I can be] both too absorbed in a task, and easily distracted” {35}*
- Unpredictable level of performance – *“My level of functioning is unpredictable, with a wide range. This makes me feel unreliable, and until recently I didn’t understand why other people could be so consistent and I couldn’t.” {46}*

To aid executive function, respondents needed extra processing time and to be free to work at their own (reasonable) pace whilst not being interrupted when performing certain tasks. Self-management of their time and tasks helped some to get the most out of their executive functioning:

*“I’m able to plan my case load to back off and switch to admin if I’m finding it hard for a few days” {53}*

The use of prompts, was suggested to aid working memory:

*“Name badges! Big writing, legible name badges for everyone. Please. Boards of photos with names and job roles underneath are also helpful, or even just having it in an email would be good.” {23}*

### 5. Change.

Change is ubiquitous in healthcare, as clinicians move between locations, jobs, specialities, and teams over the course of their careers.

*“High level of anxiety with changes in specialty, particularly as this regards getting familiar with new layouts, styles of work and familiarising with a new group of staff.” {81}*

*“I struggle when [my working environments] aren’t laid out precisely how I would need them in an emergency…it takes me a considerable amount of time to ensure everything is correctly set up.” {19}*

Participants described change being generally difficult for them, as it led to anxiety and uncertainty:

*“In my current role I have struggled with lack of structure, lack of routine, and uncertainty. Despite mentioning many times to my supervisor that I do not function well without a clear plan, and a clear structure, nothing seems to change.” {66}*

Minimising change and securing predictability in their working lives was reported to be helpful. Respondents described needing continuity where possible – e.g., in regard to their team, workplace location, or role. Where that was not possible, then reasonable notice in advance of changes helped to reduce negative impacts.

*“[I l]ocum in purely [my speciality], with regular hours that are only altered with prior agreement, clear departmental responsibilities” {81}*

*“My work was organised in blocks of the same activity, i.e., sets of home visits, telephone calls, face to face appointments.” {52}*

*“Not being sent to other clinical areas, not being sent cross site without 12 hours notice.” {30}*

*“Being told in advance of any changes to my working week” {86}*

### 6. Working environment.

The working environment impacted respondents in various ways, with sensory and people overload, overstimulation, and overwhelm reported as being challenging.

*“The hospital environment is not set up at all with autistic people in mind and can be incredibly overwhelming in terms of sensory stimulation and unpredictability. It feels like I am drifting helplessly through chaos on a daily basis when working in secondary care” {49}*

Respondents described their sensitivities in terms of bright/fluorescent lighting, smells, noise, heat, uniforms, busy environments, and open workplaces.

*“Often find that the clinical environment is too loud for me. I feel exhausted by being surrounded by people for the whole day.” {65}*

*“Lack of understanding from colleagues of the impact that, e.g., alarms or excessive light have on me. I cannot work in those environments, but I am always told that ‘it is what it is’. I find that very frustrating.” {66}*

To make hospital environments suitable for Autistic clinical professionals (and adjunctly, the Autistic patients and visitors who also utilise clinical spaces) consideration and alterations need to be afforded to lighting, noise reduction (headphones/earplugs were very popular) and sound dampening, access to quiet workspaces, and relaxation of uniform policies.

*“I would be much more productive if I had a dedicated quiet work space of my own” {73}*

*“Being able to wear comfortable clothing that don’t distract from what I’m doing - scrubs usually, but hospital policy doesn’t allow them to be worn outside theatre.” {9}*

Given that the above-mentioned environmental factors impact Autistic people so greatly, it was not surprising to find that some sense of control over their working environments was important to respondents:

*“For me, having control over my working environment and being able to set the space up effectively for optimum efficiency.” {31}*

*“I would like to have my own room to work in that is allocated and free and the start of a shift, rather than having to beg and try to find a room to work in.” {47}*

For Autistic clinical professionals, having this control, for example having one’s own office or working from home, additionally helps to minimise distraction and protect dedicated assistive technology or equipment.

*“I had screen filters but they kept being removed” {52}*

### 7. Working practises/cultures.

Some working practices and cultures negatively impacted respondents. Rotas had a large impact, as reasonable adjustment needs related to scheduling were frequently problematic. Some reported needing exemptions from particular shift patterns, a few needed flexibility, and others needed a fixed or predictable rota.

*“I struggle with inconsistent schedules.” {19}*

*“Difficulty with changing rotas, particularly night work.” {81}*

Some described managing their needs by working part time or by working on a self-employed basis, but this carried risks:

*“I am self employed. Apparently the Equality act applies to me also, I checked with [my union]! But as there is a pressure for you to see as many people as possible an hour, and they can just hire another [person] if they want, I cannot see any incentive to disclose at work as they can just not rehire me.”{47}*

When on-shift, respondents experienced pressure on their time, making them feel rushed. This left them needing more time to perform their role to the level of detail that they felt was needed. A simple adjustment of having more time for admin, catching up, or training could make a real difference.

*“Routine days are very busy with multiple issues being dealt with. Often not enough time in the day to have lunch, drink water or go to toilet” {27}*

A lack of regular break provision was commonly reported, in terms of both insufficient time allowed and a lack of provision of quiet spaces:

*“Break times in small shared spaces are challenging.” {8}*

*“I need quiet time to ‘re-energise’, this can be difficult in a busy department” {46}*

*“[I need] a space where I can briefly be alone to decompress. I often use toilet cubicles as there isn’t any other option.” {9}*

Respondents longed for an Autistic-friendly working culture. This included looking to managers for support and good team leadership but, to achieve this, managers must access training regarding Autistic employees:

*“Managers to be more aware of peoples needs and about autism and how it might impact - especially when a stereotype view of what autism is perceived and people are surprised when I tell them I am diagnosed” {41}*

Training opportunities ought not be limited to management only, indeed, colleagues ought also to be encouraged to attend:

“Colleagues and management having appropriate [knowledge], understanding and attitudes about: - about autism *including autistic colleagues/employees* and a better understanding of autism and how it means I am affected differently by things than they might be - e.g., I cope worse than them in some situations, but better in others.” {25}

Additionally, respondents wanted to be able to access support specifically tailored for Autistic clinical professionals which could provide additional input in terms of feedback, debriefing, coaching, and mentorship, as well as for administrative tasks – e.g., appraisals and revalidation activities:

*“During the time I was diagnosed, I worked with a clinical psychologist with an ASD speciality on working out my “suit of armour” for clinical work- how I manage to function in uniform. It’s the non-uniform aspects I still find difficult.” {64*}

Whilst the above has spoken to what individual employers can do, we also find it important to highlight one participant’s call for systematic change:

“*Thorough decolonisation of the postgraduate training programmes for doctors in England, including deep education and/or replacement of seniors in positions of power who do not support the need for adjustments for hidden disabilities. Only through such drastic measures at the top will the rife ableism and discrimination within the system truly stand a chance of improving to allow for real adjustments and embracement of diversity within the workforce. As it stands, some people in positions of power take tokenistic steps to outwardly appear inclusive and tick a box, whilst actively promoting ableism and discounting of hidden disabilities in their practice on the ground.”* {49}

### 8. Consequences and effects on Autistic clinical professionals.

The aforementioned challenges led respondents to feel anxious, exhausted, and burnt out. They struggled with self-doubt, rumination, and perfectionism alongside difficulties with work-life balance. These accounts emphasised the importance of employers developing an Autistic-friendly working culture.

*“I burn out often. I can’t recognise my own emotions. My colleagues usually notify me that something is wrong by showing some concern.” {46}*

*“I feel total exhaustion at the end of each clinical shift” {49}*

*“I have stopped clinical work due to burn out, now do non clinical work” {10}*

*“As junior was easily overwhelmed by too many jobs, too much noise, unclear instructions, feeling “out of control”. Could result in an out burst of frustration. Was perceived as stroppy, difficult and inflexible. Over the years, emotional intelligence training, Sim training and learning how to control my environment has made it easier.” {80}*

## Discussion

This work joins that of Curnow *et al* [14] by shining a light on the experiences of Autistic clinical professionals in relation to reasonable adjustments in the workplace. We regard our work, which highlights the clinical settings of Great Britain, as aligning with the work of Curnow *et al* [14] regarding health and education settings in Scotland, in that their findings were similar to ours; for example, discussing issues with having to ‘mask’ to suppress Autistic traits to avoid stigma, discrimination or harassment, as well as the challenges of burnout. They also reported the need for reasonable adjustments in domains including recruitment, communication, and the need for the provision of a neuro-affirming environment [14]. We agree with their findings and note that they align with Kapp [2] that challenges can arise largely from social factors and also with Shaw [1], that fitting in (or as we found ‘connecting and integrating’) is especially challenging for Autistic people. The above reported themes highlight this mismatch between Autistic clinical professionals and the external social and physical factors which directly affect them. As such, our work adds to and supports the collective picture of experiences and needs reported by Autistic clinical professionals in healthcare workplaces.

Whilst there was a lot of homogeneity, we also saw that 82 participants reported 82 different experiences. This convinced us that each Autistic person will need an individualised package of support which meets their unique needs. Sadly, the data suggest that, at least within our sample, the reasonable adjustment needs of many Autistic clinical professionals are not being met.

The legal framework provided by The Equality Act [6] is the mechanism by which disabled people are afforded protection and adaptions in Great Britain. It is social structures – such as workplaces – that have not been designed to accommodate Autistic people, which are disabling. As a society we are a long way from achieving universal design. As noted in the first quote of our first theme, an appropriate environment which would be exemplar of ‘universal design’ is especially difficult to identify, especially if individuals do not know that they are Autistic. This is particularly pertinent given that many Autistic clinicians may not realise they are Autistic (particularly due to generational differences in diagnosis). Whilst universal design would benefit Autistic clinical professionals, it would also improve patient care. However, as with all equity, diversity, inclusion, and access work, we can continually work to identify, plan, implement, and assess measures to remove disabling factors and ensure that healthcare workspaces can accommodate Autistic needs.

The 2022 Workforce Disability Equality Standard found that 4.2% of NHS staff have a disability recorded in their staff record [33]. Of those, 72.2% have adequate adjustments made by their employer to enable them to carry out their work [33] (a decrease from 76.6% in the previous year’s WDES Implementation Team [34]). Over fifty three percent of our respondents told us that they had asked for reasonable adjustments, and 34.14% had reasonable adjustments associated with being Autistic agreed with their employer. The low number of participants who have reasonable adjustments positions our survey’s responder profile as receiving proportionally fewer reasonable adjustments than disabled people overall in the same work sector in England. As we noted in this report’s introduction, another study found that 29% of Autistic doctors had not disclosed being Autistic in the workplace and only 49% of those who had asked for reasonable adjustments at work had received them [13]. Comparably, it seems that the responder profile to our survey were more willing to disclose their Autistic status to their employer, but it is unclear if all participants wanted reasonable adjustments – this might be why they did not all ask for them. Regardless of the inconsistency in findings between the Workforce Disability Equality Standard [33], Shaw *et al* [13], and this study, it can be said that disclosure is far from habitual. This might be a stance that Autistic clinical professionals have taken to protect themselves from stigma, discrimination or harassment as noted in Curnow *et al*’s work [14]; yet, not declaring Autistic status prevents employers knowing that they need to create working environments that enable their team members to work at the top of their abilities. Indeed, universal design may be additionally useful here: if workplaces were accessible by design, fewer individuals may face the challenging decision of whether to disclose.

Our responder profile’s low number of agreed reasonable adjustments could reflect selection bias, it could also indicate that there is a significant problem identifying and accessing reasonable adjustments for Autistic healthcare workers. If the latter, there are multiple possible explanations. It may be, for example, that people are less likely to disclose their Autism; that appropriate reasonable adjustments are hard to identify; that reasonable adjustments are hard or costly to implement; or that there are differences in opinion about the importance or legitimacy of reasonable adjustments for Autistic clinical professionals. This cuts two ways. First, employers might feel that requested adjustments are not reasonable. Second, in anticipation of this, Autistic clinical professionals might not ask for reasonable adjustments for fear of looking unreasonable. This risks creating a negative feedback loop, in which expectation dominates reality. This seems, to us, important and, in light of our experience and the data reported above, likely a significant factor. To illustrate briefly, it might be easily assumed that many of the challenges reported above, such as dealing with work pressures, managing conflict, communication, and busy/noisy working environments, are challenges for all people and not uniquely autistic problems – making them less likely to be viewed as legitimate targets for reasonable adjustment. However, Autistic clinical professionals carry these workplace pressures alongside the need for conscious compliance with social norms that non-Autistic people manage with comparative ease. Dealing with this is, therefore, far more burdensome for Autistic clinical professionals. The expectation that employers will not understand this difference then puts Autistic clinical profession off asking for adjustment that are and should be considered reasonable. The most obvious way to stop this negative feedback loop is open and non-judgmental communication.

Adjacently to Autistic clinical professionals’ welfare is that of Autistic people who access healthcare in Great Britain. Shaw *et al*’s [35] ‘triple empathy problem’ argues that there is a cultural divide between non-Autistic clinical professionals and their patients by virtue of the professionals’ immersion in years of clinical culture and training. Research consistently shows that Autistic people have a reduced life expectancy and live with significantly higher rates of co-occurring health conditions [36]. We also know that barriers to healthcare may lead to adverse health outcomes [37]. Therefore, an added value of an inclusive workforce approach is that Autistic-friendly working environments, which directly benefit Autistic clinical professionals will also indirectly benefit Autistic patients. When Autistic clinical professionals are supported by reasonable adjustments, with an associated increase in understanding of Autism within their teams, their increased retention and representation may increase recognition of Autistic patients. Essentially, maintaining and supporting diversity in the healthcare workforce is good for patients too.

The findings of this study might have the potential to benefit healthcare more generally in terms of facilitating recognition of, and acting upon, the needs of Autistic employees to improve environments for both Autistic workforces and those Autistic people who will be treated in them. Considerations could be made by those in power of how practices, environments, demands, and support could be addressed and improved for those who work in and access healthcare in Great Britain. To inform employers, clinicians, patients, and policymakers better, this pilot work would need to be expanded into a larger piece of research to better represent the diverse population that constitute the Autistic workforce. Also, more broadly, and as also noted by Curnow *et al* [14] there needs to be enquiry of the intersection of other experiences equally qualifying for reasonable adjustments, for example: those experiences which commonly co-occur under the umbrella of neurodivergence, such as Dyslexia, Dyspraxia, Tourette Syndrome, etc [38].

## Limitations

This work only represents the responder profile and is not representative of all Autistic clinical professionals in Great Britain. It is not known how many Autistic clinical professionals there are in practise and there are multiple unknown variables which affect Autistic people being identified in society (e.g., under/over-diagnosis). Furthermore, we have not included every clinical profession (e.g., dentists), or others supporting healthcare. Additionally, our recruitment strategy would have excluded those who are less/not active on social media.

The fully anonymised data collection design meant that there was no way to validate the accuracy of the information, avoid duplicate submissions, and avoid participation from ineligible respondents.

Specific data on socioeconomic status and educational attainment levels were not recorded, however it can be noted that registration with General Medical Council, Nursing and Midwifery Council, and Health & Care Professions Council is dependent upon successfully completing university education.

## Conclusion

In this paper we have reported our findings from survey data given to us by eighty-two Autistic clinical professional survey respondents. We reported the 8 themes identified in our analysis which informed us of Autistic clinical professionals’ reasonable adjustment needs, what reasonable adjustments they had in place, and the barriers to reasonable adjustment adoption in their workplaces in Great Britain.

We were struck by and applaud the tenacity of Autistic clinical professionals in their service to others despite the many challenges and barriers that they described; however, the resilience of Autistic clinical professionals must not be relied upon solely to keep them in practice.

### To Autistic clinical professionals

This work aimed to aid you to not feel alone: it is possible to get support in the workplace. We hope that you find this work useful in starting conversations with your employer about what it means to be Autistic and the variety of reasonable adjustments which can be adopted to help you to continue to contribute to, and shine in, health services.

### To employers

We recognise that adjustments need to be *reasonable* and that means working within the resources available, but taking steps towards creating an Autism-friendly working environment will help you to meet your obligations to the Equality Act. It will additionally pay dividends by helping to attract and retain skilled Autistic clinical professionals to benefit your service.

This report shows that each Autistic clinical professional is unique, and each will need a different package of adjustments to meet their needs. We agree with Davis *et al*’s [39] findings that Autistic clinical professionals ought not be left to struggle to identify, establish, and request reasonable adjustments from their employers; rather that employers should actively engage in identification and implementation of adjustments that would benefit Autistic clinical professionals, employers, and the healthcare services that they collectively offer. We offer only a small snapshot of the variety of potential adjustments that could be made for Autistic clinical professionals. We encourage employers to learn about Autism, acknowledge differences, think about and discuss workplace needs with their Autistic clinical professionals, be flexible where needed, and iteratively work with team members towards the goal of enabling *all* to equitably contribute to their professions.

## Supporting information

S1 File S1_Survey questions. (DOCX)

## References

[1] Shaw SCK. The impacts of dyslexia and dyspraxia on medical education. Doctoral Thesis, University of Brighton and the University of Sussex. 2021 [cited 2024 Sep 14]. Available from: https://cris.brighton.ac.uk/ws/portalfiles/portal/25765918/Shaw_Thesis_.pdf

[2] Pellicano E, Fatima U, Hall G, Heyworth M, Lawson W, Lilley R, et al. A capabilities approach to understanding and supporting autistic adulthood. Nat Rev Psychol. 2022;1(11):624–39. doi: 10.1038/s44159-022-00099-z 36090460 PMC9443657

[3] Kapp SK. Autistic community and the neurodiversity movement: stories from the frontline. Singapore: Palgrave Macmillan Singapore. 2020. doi: 10.1007/978-981-13-8437-0

[4] National Institute for Health and care Excellence. Autism in adults: How common is it? [online]. 2024 [cited 2024 Sep 14]. Available from: https://cks.nice.org.uk/topics/autism-in-adults/background-information/prevalence/

[5] Maenner MJ, Warren Z, Williams AR, Amoakohene E, Bakian AV, Bilder DA, et al. Prevalence and characteristics of autism spectrum disorder among children aged 8 years - autism and developmental disabilities monitoring network, 11 sites, United States, 2020. MMWR Surveill Summ. 2023;72(2):1–14. doi: 10.15585/mmwr.ss7202a1 36952288 PMC10042614

[6] The Equality Act 2010 c.15 [cited 2024 Sep 14]. Available from: https://www.legislation.gov.uk/ukpga/2010/15/contents

[7] The Advisory, Conciliation and Arbitration Service (ACAS). Reasonable adjustments at work [online]. 2022 [cited 2024 Sep 14]. Available from: https://www.acas.org.uk/reasonable-adjustments

[8] Professional Standards Authority for Health and Social Care. Decisions about practitioners [online]. N.d. [cited 2024 Sep 14]. Available from: https://www.professionalstandards.org.uk/what-we-do/our-work-with-regulators/decisions-about-practitioners

[9] General Medical Council. Welcomed and valued. 2024. [cited 2024 Sep 14]. Available from: https://www.gmc-uk.org/education/standards-guidance-and-curricula/guidance/welcomed-and-valued

[10] Nursing and Midwifery Council. Understanding Fitness to Practise: Health. 2017 [cited 2024 Sep 14]. Available from: https://www.nmc.org.uk/ftp-library/understanding-fitness-to-practise/fitness-to-practise-allegations/health/

[11] Doherty M, Johnson M, Buckley C. Supporting autistic doctors in primary care: challenging the myths and misconceptions. Br J Gen Pract. 2021;71(708):294–5. doi: 10.3399/bjgp21X716165 34319879 PMC8249017

[12] McCowan S, Shaw SCK, Doherty M, Grosjean B, Blank P, Kinnear M. A full CIRCLE: inclusion of autistic doctors in the Royal College Of Psychiatrists’ values and Equality Action Plan. Br J Psychiatry. 2022;221(1):371–3. doi: 10.1192/bjp.2022.14 35152922

[13] Shaw SCK, Fossi A, Carravallah L, Rabenstein K, Ross W, Doherty M. The experiences of autistic doctors: a cross-sectional study. Front Psychiatry. 2023;14:1160994. doi: 10.3389/fpsyt.2023.1160994 37533891 PMC10393275

[14] Curnow E, Rutherford M, Maciver D, Johnston L, Utley I, Murray M, et al. Beyond accommodations: Supporting autistic health professionals. National Autism Implementation Team (NAIT), Edinburgh, UK. 2024. [cited 2024 Sep 14]. Available from: https://www.thirdspace.scot/wp-content/uploads/2024/03/Beyond-Accommodations-Supporting-Autistic-Health-Professionals.pdf

[15] den Houting J. Neurodiversity: An insider’s perspective. Autism. 2019;23(2):271–3. doi: 10.1177/1362361318820762 30556743

[16] Ives J. A method of reflexive balancing in a pragmatic, interdisciplinary and reflexive bioethics. Bioethics. 2014;28(6):302–12. doi: 10.1111/bioe.12018 23444909

[17] Bryman A. Social research methods (5th ed.). Oxford: Oxford University Press. 2016.

[18] Lockwood Estrin G, Milner V, Spain D, Happé F, Colvert E. Barriers to autism spectrum disorder diagnosis for young women and girls: a systematic review. Rev J Autism Dev Disord. 2021;8(4):454–70. doi: 10.1007/s40489-020-00225-8 34868805 PMC8604819

[19] Doyle N, Medhurst B. Evaluating and supporting Neurodifferences* at work. [online] London: The Society of Occupational Medicine. 2022. Available from: https://www.som.org.uk/sites/som.org.uk/files/Evaluating_and_supporting_Neurodifferences_at_work_March_2022.pdf

[20] Smith H, Manzini A, Ives J. Inclusivity in TAS research: an example of EDI as RRI. J Responsible Technol. 2022;12:100048. doi: 10.1016/j.jrt.2022.100048

[21] Pellicano E, Adams D, Crane L, Hollingue C, Allen C, Almendinger K, et al. Letter to the Editor: a possible threat to data integrity for online qualitative autism research. Autism. 2024;28(3):786–92. doi: 10.1177/13623613231174543 37212144

[22] Shaw SCK. An introduction to the role of relational ethics in qualitative healthcare education research. Nurse Educ Pract. 2019;36:157–8. doi: 10.1016/j.nepr.2019.04.005 30986661

[23] Braun V, Clarke V. Using thematic analysis in psychology. Qualitative Research in Psychology. 2006;3(2):77–101. doi: 10.1191/1478088706qp063oa

[24] Braun V, Clarke V. Thematic analysis: a practical guide. London: Sage. 2022.

[25] Hsieh H-F, Shannon SE. Three approaches to qualitative content analysis. Qual Health Res. 2005;15(9):1277–88. doi: 10.1177/1049732305276687 16204405

[26] NHS England and NHS Improvement. Gender pay gap report 2022: A combined report for NHS England and NHS Improvement [online]. 2023 [cited 2024 Sep 14]. Available from: https://www.england.nhs.uk/long-read/gender-pay-gap-report-2022/

[27] NHS Workforce and Business. NHS workforce [online]. 2023 [cited 2024 Sep 14]. Available from: https://www.ethnicity-facts-figures.service.gov.uk/workforce-and-business/workforce-diversity/nhs-workforce/latest/

[28] Autistic Doctors International. [online]. 2024 [cited 2024 Sep 14]. Available from: https://autisticdoctorsinternational.com/

[29] UK Government. Access to work: get support if you have a disability or health condition. [online]. 2023 [cited 2024 Sep 14]. Available from: https://www.gov.uk/access-to-work

[30] World Health Organisation. Surgical Safety Checklist [online]. 2009 [cited 2024 Sep 14]. Available from: https://www.who.int/teams/integrated-health-services/patient-safety/research/safe-surgery/tool-and-resources

[31] Belcher H. Autistic people and masking. National Autistic Society [online]. 2022 [cited 2024 Sep 14]. Available from: https://www.autism.org.uk/advice-and-guidance/professional-practice/autistic-masking

[32] Center on the Developing Child. Executive Function & Self-Regulation. Harvard University. [online]. 2023 [cited 2024 Sep 14]. Available from: https://developingchild.harvard.edu/science/key-concepts/executive-function/

[33] WDES Implementation Team. Workforce Disability Equality Standard 2022 data analysis report for NHS trusts and foundation trusts [online]. 2023 [cited 2024 Sep 14]. Available from: https://www.england.nhs.uk/long-read/workforce-disability-equality-standard-2022-data-analysis-report-for-nhs-trusts-and-foundation-trusts/

[34] WDES Implementation Team. Workforce Disability Equality Standard: 2021 data analysis report for NHS trusts and foundation trusts [online]. 2022 [cited 2024 Sep 14]. Available from: https://www.england.nhs.uk/publication/workforce-disability-equality-standard-2021-data-analysis-report-for-nhs-trusts-and-foundation-trusts/

[35] Shaw SC, Carravallah L, Johnson M, O’Sullivan J, Chown N, Neilson S, et al. Barriers to healthcare and a “triple empathy problem” may lead to adverse outcomes for autistic adults: A qualitative study. Autism. 2024;28(7):1746–57. doi: 10.1177/13623613231205629 37846479 PMC11191657

[36] O’Nions E, Lewer D, Petersen I, Brown J, Buckman JEJ, Charlton R, et al. Estimating life expectancy and years of life lost for autistic people in the UK: a matched cohort study. Lancet Reg Health Eur. 2023;36:100776. doi: 10.1016/j.lanepe.2023.100776 38188276 PMC10769892

[37] Doherty M, Neilson S, O’Sullivan J, Carravallah L, Johnson M, Cullen W, et al. Barriers to healthcare and self-reported adverse outcomes for autistic adults: a cross-sectional study. BMJ Open. 2022;12(2):e056904. doi: 10.1136/bmjopen-2021-056904 PMID: 35193921 PMC8883251

[38] Doyle N. Neurodiversity at work: a biopsychosocial model and the impact on working adults. Br Med Bull. 2020;135(1):108–25. doi: 10.1093/bmb/ldaa021 32996572 PMC7732033

[39] Davies J, Heasman B, Livesey A, Walker A, Pellicano E, Remington A. Autistic adults’ views and experiences of requesting and receiving workplace adjustments in the UK. PLoS One. 2022;17(8):e0272420. doi: 10.1371/journal.pone.0272420 35930548 PMC9355205

