# Correction: Reasonable adjustments for autistic clinicians: A qualitative study

**DOI:** 10.1371/journal.pone.0325000

**Published:** 2025-05-20

**Authors:** The PLOS One Staff

The ORCID iDs are missing for the second and fourth author. Please see the authors’ respective ORCID iDs here:

- Author Sebastian Shaw’s ORCID iD is: 0000-0001-9597-7436 (https://orcid.org/0000-0001-9597-7436).
- Author Jonathan Ives’s ORCID iD is: 0000-0002-5233-5000 (https://orcid.org/0000-0002-5233-5000).

The publisher apologizes for the error.
