# Supplementary material for: Reasonable adjustments for autistic clinicians: A qualitative study
Source: PLoS One. 2025 Mar 25;20(3):e0319082. doi: 10.1371/journal.pone.0319082 (PMC11936167; doi:10.1371/journal.pone.0319082)
Supplement: S1 File — (DOCX) [file pone.0319082.s001.docx]

**Survey Questions**

1. Participant information
   1. I have read this
2. Consent information
   1. I have read, agree to, and consent to the above
3. I confirm that I am autistic.
   (Self-diagnosed autistic is fine. You do not need to be formally diagnosed as autistic to enrol in this study.)
   1. Yes
   2. No
      1. Lived autistic experience is critical to participate in this survey.
         We thank you for your interest in this survey, but if you do not have this experience then we cannot invite you to participate in this survey.
4. Self-diagnosed or formal diagnosis? (You do not need to be formally diagnosed as autistic to enrol in this study.)
   1. Self-diagnosed
   2. Formal diagnosis
5. Demographic information: We’d like this survey to reflect as diverse a population as possible. We would like to note, report, and highlight if there are differences in reasonable adjustments provided to people as related to their characteristics of age, gender, ethnicity, or sexual orientation.
   1. How old are you please?
      1. 18-29
      2. 30-39
      3. 40-49
      4. 50-59
      5. 60-69
      6. 70-79
      7. 80-89
      8. 90-99
      9. Prefer not to say
   2. Please describe your gender or enter n/a if you prefer not to say
   3. Please describe your ethnicity or enter n/a if you prefer not to say
   4. Please describe your sexual orientation or enter n/a if you prefer not to say
6. Which clinical professional body are you registered with?
   1. Tick box: GMC/NMC/HCPC
7. What is your role? (e.g. nurse, doctor, paramedic, etc.)
8. Which clinical speciality do you work in? (e.g., critical care, primary care etc.)
9. Which environment do you work in? (tertiary hospital, community outpatients, etc.)
10. Are you employed by:
    1. A public healthcare provider, e.g. NHS
    2. A private healthcare provider, e.g. private hospital
    3. Other (e.g., agency, self-employed).
       1. Please describe your employer (e.g. agency, self-employed)
11. Does your experience of being autistic raise challenges in your clinical work?
    1. Yes
       1. Please describe the challenges that you face resulting from being autistic in the workplace?
    2. No
       1. Could you please tell us more about your answer? For example:
          Do you have no issues at all in the workplace as an autistic person?
          Do you have reasonable adjustments that remove challenges?
12. Were you recognised as autistic (either you realised yourself, or others realised) as a child or as an adult (i.e. 18+ years old)?
    1. As a child (before 18 years old)
       1. If you were recognised as autistic as a child, did you receive any accommodations for this? (e.g., formally via an Education Health and Care Plan, or informally via home schooling).
          *We recognise that participants may have reasonable adjustments for more than one disability; please answer only for those accommodations that were in place as related to your autistic status.*
          1. Yes
             1. Did these accommodations help you to recognise what you needed when you transitioned from education to the workplace?

Yes

Could you please tell us more about your answer? For example, were your educational accommodations also relevant to your workplace?

No

Could you please tell us more about your answer? For example, were your educational accommodations not relevant to your workplace?

- - - 1. No
  1. As an adult (18+)

1. As an autistic person, what reasonable adjustments do you feel you need for your clinical work? *We recognise that participants may have reasonable adjustments for more than one issue; please answer only about those adjustments that make your workplace accessible as per your autistic status.*
2. Have you asked for reasonable adjustments from your employer?
   1. Yes
   2. No
      1. Why have you not asked for reasonable adjustments from your employer?
3. Do you have reasonable adjustments associated with being autistic agreed with your employer?
   1. Yes
      1. What reasonable adjustments related to being autistic do you have in place for your work as a clinician?
         *You might have reasonable adjustments which purely address being autistic.*
         *Alternatively, you might have reasonable adjustments that help with another issue that you have, but also happen to address a need that you have due to being autistic.*
      2. Were these adjustments (tick all that apply):
         1. Formally/informally arranged?
         2. Organised yourself?
         3. Organised by your employer?
         4. Organised jointly between you and your employer?
         5. Assessed by others and then agreed and adopted for you by your employer?
         6. Something else?
      3. Would you like to tell us more about your experience of your reasonable adjustments being arranged?
   2. No
4. Has Occupational Health made formal recommendations for reasonable adjustments related to being autistic for your clinical workplace?
   1. Yes
      1. Were Occupation Health's recommendations implemented?
         1. Implemented
            1. Would you like to tell us more about your experiences with Occupational Health regarding reasonable adjustments related to being autistic?
         2. Not implemented
            1. Would you like to tell us more about your experiences with Occupational Health regarding reasonable adjustments related to being autistic?
         3. Some were implemented
            1. Would you like to tell us more about your experiences with Occupational Health regarding reasonable adjustments related to being autistic?
   2. No
      1. Would you like to tell us more about your experiences with Occupational Health regarding reasonable adjustments related to being autistic?
5. Whether you have reasonable adjustments in your workplace or not, are there other reasonable adjustments related to being autistic that you need but do not have?
   1. Yes
      1. What reasonable adjustments related to being autistic do you need but not have?
   2. No
6. Have you experienced barriers to getting the reasonable adjustments that you need?
   1. Yes
      1. What is/was the barrier?
      2. What would be needed for that barrier(s) to be removed?
   2. No
7. Is there anything else you would like to tell us about your experiences as an autistic clinician regarding reasonable adjustments?
8. You are welcome to submit your responses to us now, but please remember, once you press submit, it will not be possible to remove your responses from the dataset.
   By submitting you are consenting for us to your use your data in the way outlined in the participant information and consent information at the top of this survey (Please see here for the link to the PDF for participant and consent info https://www.bristol.ac.uk/population-health-sciences/centres/ethics/research/reasonable-adjustments-for-autistic-clinicians-raac/)
   If you do not want to add your responses to this survey’s dataset, please just close this window now, none of your responses will be recorded, and we thank you for your consideration of this survey.
   If you are happy to add your responses to this survey’s dataset, please press the ‘submit’ button now, and we thank you for your participation.
